# Deciphering the molecular landscape of ionising radiation-induced eye damage with the help of genomic data mining

**DOI:** 10.2478/aiht-2024-75-3817

**Published:** 2024-06-29

**Authors:** Katarina Baralić, Predrag Božović, Danijela Đukić-Ćosić

**Affiliations:** University of Belgrade, Faculty of Pharmacy, Department of Toxicology “Akademik Danilo Soldatović“, Belgrade, Serbia; University of Belgrade Vinča Institute of Nuclear Sciences, Department of Radiation and Environmental Protection, Belgrade, Serbia

**Keywords:** data mining, DNA damage, miRNAs, ocular damage, radiation health effects, istraživanje podataka, oštećenje DNA, miRNAs, oštećenje oka, biološki učinci zračenja

## Abstract

Even at low levels, exposure to ionising radiation can lead to eye damage. However, the underlying molecular mechanisms are not yet fully understood. We aimed to address this gap with a comprehensive *in silico* approach to the issue. For this purpose we relied on the Comparative Toxicogenomics Database (CTD), ToppGene Suite, Cytoscape, GeneMANIA, and Metascape to identify six key regulator genes associated with radiation-induced eye damage (*ATM*, *CRYAB*, *SIRT1*, *TGFB1*, *TREX1*, and *YAP1*), all of which have physical interactions. Some of the identified molecular functions revolve around DNA repair mechanisms, while others are involved in protein binding, enzymatic activities, metabolic processes, and post-translational protein modifications. The biological processes are mostly centred on response to DNA damage, the p53 signalling pathway in particular. We identified a significant role of several miRNAs, such as hsa-miR-183 and hsamiR-589, in the mechanisms behind ionising radiation-induced eye injuries. Our study offers a valuable method for gaining deeper insights into the adverse effects of radiation exposure.

## Sažetak

Izloženost ionizirajućem zračenju čak i pri niskim razinama može pridonijeti nastanku oštećenja oka. Međutim, osnovni molekulski mehanizmi i dalje nisu potpuno razjašnjeni. Cilj našega istraživanja bio je ispuniti tu nedostajuću kariku primjenom sveobuhvatnog *in silico* pristupa problemu. U tu svrhu, pomoću genomskih baza podataka, portala i poslužitelja (Comparative Toxicogenomics Database, ToppGene Suite portal, Cytoscape, GeneMANIA i Metascape), identificirano je šest ključnih regulacijskih gena koji su povezani s oštećenjem oka prouzročenog ionizirajućim zračenjem (*ATM*, *CRYAB*, *SIRT1*, *TGFB1*, *TREX1* i *YAP1*) i koji su svi bili u fizičkoj interakciji. Neke od identificiranih molekulskih funkcija odnosile su se na mehanizme popravka oštećenja DNA, a druge su bile uključene u vezanje proteina, enzimsku aktivnost, metaboličke procese i posttranslacijske modifikacije proteina. Biološki procesi uglavnom su bili povezani s odgovorom na oštećenje DNA, pogotovo sa signalnim putem p53. Uočena je i značajna uloga nekoliko miRNA, poput hsa-miR-183 i hsa-miR-589, u mehanizmima povezanima s oštećenjem oka prouzročenog ionizirajućim zračenjem. Osim toga, u ovom je istraživanju opisana korisna metoda za ispitivanje štetnih učinaka izloženosti zračenju.

Recent years have seen a significant increase in the use of radiation-emitting materials, devices, and ionising radiation technology, particularly in sectors such as industry, agriculture, and medicine (1). With the widespread adoption of interventional radiology procedures worldwide, workers involved in these operations may face substantial levels of radiation exposure due to the complexity and duration of each procedure (2). According to the United Nations Scientific Committee on the Effects of Atomic Radiation (UNSCEAR), about 4.2 billion medical procedures were performed between 2009 and 2018, including 24 million interventional radiology procedures, involving around 11 million workers between 2010 and 2014 (3).

The International Commission on Radiological Protection (ICRP) Report 103 (4) recommended a review of non-cancerous effects of ionising radiation on normal tissues at low doses, including the sensitivity of the eye to radiation (5). The development of cataracts was previously considered a common tissue reaction, with effective dose thresholds established by the ICRP in 2007 at 5 Sv for chronic exposures and 2 Sv for acute exposures (4, 6). As for the absorbed dose, based on new epidemiological evidence, the ICRP has lowered the dose threshold for the eye lens to 0.5 Gy, having taken into account the latency period and the possibility of cataracts occurring at much lower doses, particularly with chronic exposure to relatively small doses. Consequently, the annual effective dose limit for the eye lens has been lowered from 150 mSv to 20 mSv (7).

Cardiologists are estimated to receive an average cumulative dose of 6 Sv without modifications to personal protective equipment, while support staff may receive around 1.5 Sv (8, 9). Moreover, a study tracking over 35,000 radiological technicians for 20 years revealed that even a relatively low cumulative dose of up to 60 mGy throughout their working lives could induce radiation injuries and elevate the risk of cataract development (10, 11), impaired vision and, ultimately, blindness as significant ocular adverse effects associated with exposure to ionising radiation (12, 13). Radiation retinopathy, on the other hand, presents as a progressive series of vascular changes, primarily affecting the macula. The onset, progression, and severity of retinopathy are mainly determined by the total radiation dose and treatment schedule, although factors such as concurrent chemotherapy and pre-existing diabetes may exacerbate vasculopathy by intensifying the attack of oxygen-derived free radicals on vascular cells (14).

However, the mechanisms underlying radiation-induced eye damage remain insufficiently understood. In Serbia, ethical concerns and considerations for animal welfare prohibit radiation testing on animals (15). Alternative methods, such as *in silico* testing, which employs computational methods and data analysis, could therefore be used instead to investigate the effects of radiation exposure. Online resources compiling information on various stressors and gene expression changes potentially contributing to the pathogenesis of different diseases can facilitate data mining, analysis, and discussion of observed associations (16). Toxicogenomics data mining, focusing on the effects of chemicals on genes and gene expression patterns (17), can also be applied to different stressors, including radiation. By utilising the existing databases like the Comparative Toxicogenomics Database (CTD), genes affected by radiation exposure can be identified to gain insights into activated or disrupted molecular responses and pathways (18,19,20).

Taking all of this into consideration, the primary aim of our study was to explore the mechanisms of radiation-induced eye injury using gene databases, software, and tools. Additionally, we aimed to demonstrate the utility of these resources in effectively identifying the effects and causes of damage resulting from radiation exposure.

## MATERIALS AND METHODS

**Figure 1:**
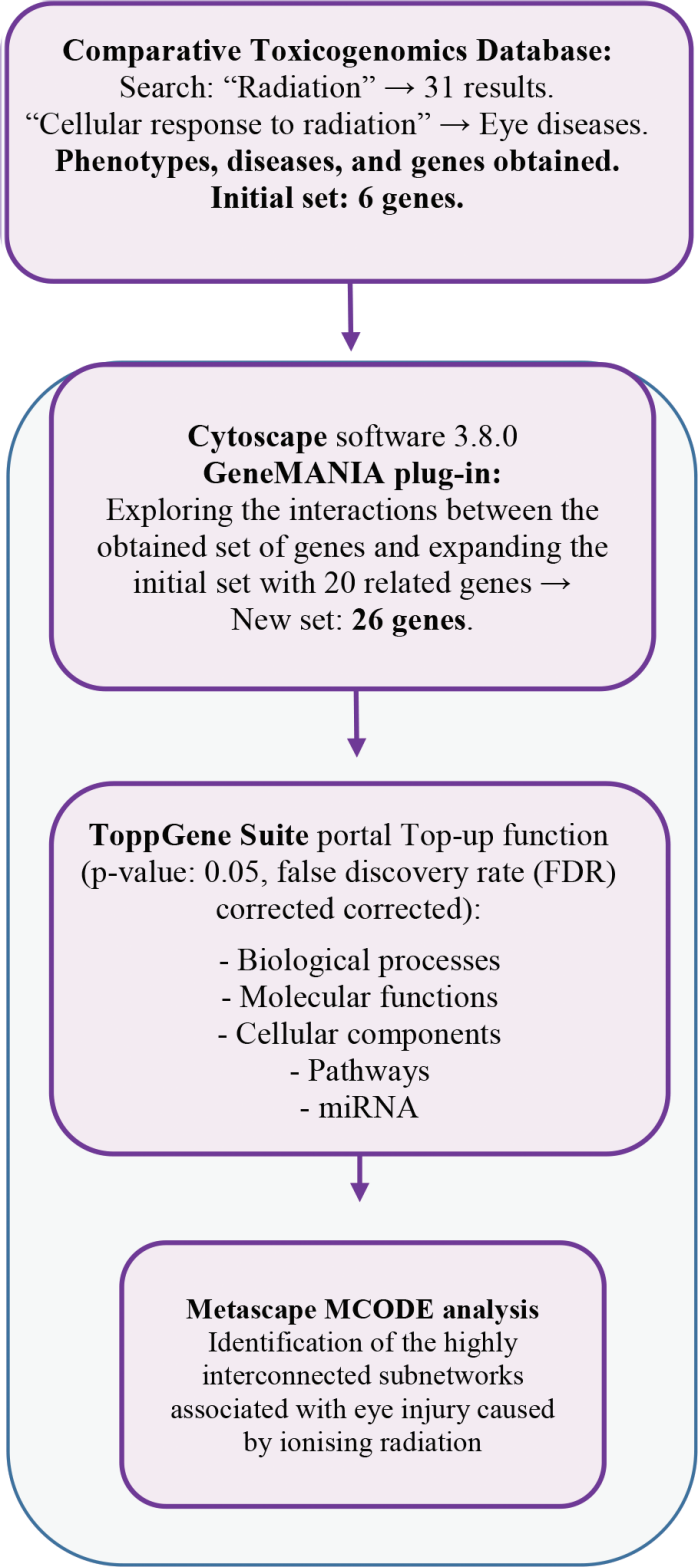
Detailed step-by-step diagram showing different phases of gene database analysis applied to investigate the relationship between the ionising radiation and eye injury

Data presented in this article were obtained in July 2023. Figure 1 shows all the steps of the applied bioinformatics analysis explained in detail later in the text.

### Comparative Toxicogenomics Database

The Comparative Toxicogenomics Database (CTD; *https://ctdbase.org*), more and more often referred to as the “Golden Set Database”, is a publicly available database that evaluates and summarises the data on associations between chemicals, genes, and diseases, and also provides information about the gene ontology (biological processes, molecular functions), molecular pathways, and phenotype (21, 22). It is updated regularly to ensure that all the information it contains is reliable, consistent, and easily accessible. In our study, we relied on CTD to assess cellular response to ionising radiation and identify a set of genes linked to the eye injury based on the listed diseases and interacting genes. Using the key word “radiation” to search through gene ontology (GO) annotations, we obtained 31 matches. Among these, “cellular response to radiation” was the broadest term encompassing all radiation types in the CTD database. From the available options we selected “response to ionising radiation”, as this gene ontology term covers all types of ionising radiation, including cellular response to gamma radiation and X-rays. Then we filtered diseases of the eye associated with “response to ionising radiation” (“eye diseases”) and identified six genes associated with the listed eye diseases for further analysis. Our decision to consider all listed eye diseases was driven by a wish to gain a deeper and more comprehensive insight into the effects of ionising radiation on eye health across a range of clinical conditions.

### GeneMANIA Cytoscape plug in

The GeneMANIA Cytoscape plug-in (*https://apps.cytoscape.org/apps/genemania*) generates a list of the genes most similar to the ones entered into the query and explores the links between them (23). We used this plug-in to explore gene interactions, but also to expand the original set with 20 related genes [test organism: *H. sapiens* (human)]. The obtained gene set for further analysis now consisted of 26 genes. There are several possible interactions identified by the GeneMANIA server: protein-protein interactions, co-expressions (where gene expression levels show similarity across conditions in a gene expression study), gene interactions (functional associations observed when one gene disruption affects the other gene), shared protein domains, co-localisation (genes expressed in the same tissue or proteins found in the same location), pathway sharing (gene products participate in the same reaction within a pathway), and finally, predicted functional relationships, most often protein interactions, derived from known functional relationships from another organism based on orthology (two proteins are predicted to interact if their orthologues are known to interact in that organism) (24).

### ToppGene Suite portal

ToppGene Suite (*https://toppgene.cchmc.org*) is an online tool that uses functional descriptors and a protein association network to prioritise candidate genes. The ToppGene ToppFun feature (*https://toppgene.cchmc.org/enrichment.jsp*) allows exploration of ontologies (gene ontology, pathway), phenotype, pharmacome, miRNA, and other parameters (25). In this study, the ToppGeneSuite portal (ToppFun function) was used to identify probable molecular mechanisms involved in radiation-associated eye injury based on the above mentioned set of 26 genes to better understand their role in the context of a larger biological system. Biological processes, molecular functions, cellular components, molecular pathways, and miRNA were selected as the main functions of interest [p-value: 0.05, corrected for false discovery rate (FDR)]. The obtained miRNA were ranked by the mirSVR, a new machine learning method for ranking microRNA target sites by a down-regulation score (26).

### Metascape

Metascape (*https://metascape.org*) is a web-based portal and a comprehensive resource for gene list annotation and analysis (27) which uses the Molecular Complex Detection (MCODE) algorithm to locate dense gene clusters in a network based on their topology (27, 28). We used this algorithm to identify highly interconnected subnetworks of the identified genes and related biological processes or diseases of interest. An MCODE network contains a subset of proteins that form physical interactions with at least one other member in the list. If the network contains between three and 500 proteins, the algorithm is applied to identify densely connected network components (27).

To construct the figures, the obtained network was downloaded from Metascape and adjusted in the Cytoscape software [i.e. extracted from the pre-constructed GeneMANIA network by the Cytoscape MCODE plug-in (*https://apps.cytoscape.org/apps/mcode*)].

## RESULTS

Table 1 shows 13 eye diseases and six genes (*ATM*, *CRYAB*, *SIRT1*, *TGFB1*, *TREX1*, and *YAP1*) associated with ionising radiation. Some diseases are repeated because of their association with different phenotypes (e.g., cataract as cellular response to gamma radiation and X-ray). Having in mind that gene inference network may differ depending on the gene ontology process listed in the phenotype column, all of the repeats have been listed together with the corresponding genes.

**Table 1:** Diseases and interacting genes associated with eye injury caused by radiation (*CTD; http://ctdbase.org/*)

**Phenotype**	**Disease**	**Gene**
Cellular response to gamma radiation	Cataract	*ATM | CRYAB*
Cellular response to ionising radiation	Diabetic retinopathy	*SIRT1*
Cellular response to ionising radiation	Retinal diseases	*SIRT1*
Cellular response to gamma radiation	Coloboma, ocular, with or without hearing impairment, cleft lip/palate, and/or impaired intellectual development	*YAP1*
Cellular response to gamma radiation	Cataract 16, multiple types	*CRYAB*
Regulation of cellular response to gamma radiation	Cataract	*ATM*
Cellular response to gamma radiation	Myopathy, myofibrillar, fatal infantile hypertonic, alpha-B crystallin-related	*CRYAB*
Cellular response to X-ray	Cataract	*ATM*
Cellular response to gamma radiation	Alpha-B crystallinopathy	*CRYAB*
Cellular response to ionising radiation	Dry eye syndromes	*TGFB1*
Cellular response to ionising radiation	Cataract	*ATM*
Cellular response to gamma radiation	Vasculopathy, retinal, with cerebral leukodystrophy	*TREX1*
Cellular response to ionising radiation	Graves disease	*TGFB1*

**Figure 2:**
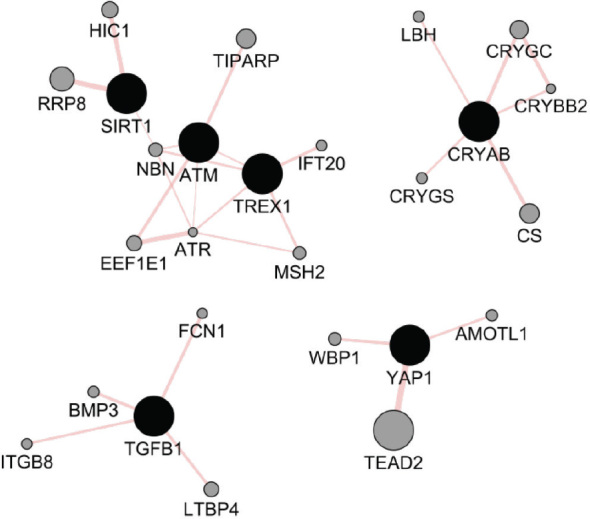
GeneMANIA network of genes associated with eye injury caused by ionising radiation (black) together with the 20 related genes (grey). Interaction type: 100 % physical interactions (GeneMANIA; *https://apps.cytoscape.org/apps/genemania*)

Table 2 shows the expanded gene set, including the six genes from the original query and 20 related genes. The interactions for all 26 genes were physical (Figure 2), indicating that they might be involved in the same biological processes or pathways and that their products may interact to carry out specific functions.

**Table 2:** Gene set linked to the eye injury caused by ionising radiation based on CTD and GeneMANIA analysis (*http://ctdbase.org/*; *https://apps.cytoscape.org/apps/genemania*)

**Gene symbol**	**Gene name**	**Gene ID**
*ATM*	ATM serine/threonine kinase	472
*CRYAB*	Crystallin alpha B	1410
*SIRT1*	Sirtuin 1	23411
*TGFB1*	Transforming growth factor beta 1	7040
*TREX1*	Three prime repair exonuclease 1	11277
*YAP1*	Yes1 associated transcriptional regulator	10413
*TEAD2*	TEA domain transcription factor 2	8463
*RRP8*	Ribosomal RNA processing 8	23378
*CS*	Citrate synthase	1431
*TIPARP*	TCDD inducible poly(ADP-ribose) polymerase	25976
*CRYGC*	Crystallin gamma C	1420
*HIC1*	HIC ZBTB transcriptional repressor 1	3090
*EEF1E1*	Eukaryotic translation elongation factor 1 epsilon 1	9521
*LTBP4*	Latent transforming growth factor beta binding protein 4	8425
*NBN*	Nibrin	4683
*MSH2*	MutS homolog 2	4436
*WBP1*	WW domain binding protein 1	23559
*AMOTL1*	Angiomotin like 1	154810
*BMP3*	Bone morphogenetic protein 3	651
*LBH*	LBH regulator of WNT signaling pathway	81606
*CRYGS*	Crystallin gamma S	1427
*IFT20*	Intraflagellar transport 20	90410
*ITGB8*	Integrin subunit beta 8	3696
*FCN1*	Ficolin 1	2219
*CRYBB2*	Crystallin beta B2	1415
*ATR*	ATR serine/threonine kinase	545

Table 3 shows the top 15 gene ontology (molecular functions, biological processes, cellular components) and molecular pathways listed by statistical significance.

**Table 3:** Top 15 gene ontology (molecular functions, biological processes) and molecular pathways associated with eye injury caused by ionising radiation (*https://toppgene.cchmc.org*)

	**ID**	**Name**	**p-value**	**Input genes**	**Annotated genes**
Molecular functions	GO:0005212	structural constituent of eye lens	2.806E-8	4	25
	GO:0032405	MutLalpha complex binding	6.840E-8	3	7
	GO:0032404	mismatch repair complex binding	3.214E-7	3	11
	GO:0032407	MutSalpha complex binding	3.412E-5	2	7
	GO:0044877	protein-containing complex binding	2.123E-4	9	1726
	GO:0003950	NAD^+^ ADP-ribosyltransferase activity	6.938E-4	2	30
	GO:0047485	protein N-terminus binding	7.323E-4	3	137
	GO:0050699	WW domain binding	8.919E-4	2	34
	GO:0004108	citrate (Si)-synthase activity	1.304E-3	1	1
	GO:0160011	NAD-dependent protein decrotonylase activity	1.304E-3	1	1
	GO:0160012	NAD-dependent histone decrotonylase activity	1.304E-3	1	1
	GO:0036440	citrate synthase activity	1.304E-3	1	1
	GO:0016763	pentosyltransferase activity	2.491E-3	2	57
	GO:0106231	protein-propionyllysine depropionylase activity	2.603E-3	1	2
	GO:0032129	histone deacetylase activity (H3-K9 specific)	2.603E-3	1	2
Biological processes	GO:0071479	cellular response to ionising radiation	1.415E-11	7	90
	GO:0042770	signal transduction in response to DNA damage	8.815E-11	8	200
	GO:0010212	response to ionising radiation	9.545E-11	8	202
	GO:0072331	signal transduction by p53 class mediator	9.545E-11	8	202
	GO:0071480	cellular response to gamma radiation	5.615E-10	5	34
	GO:0030330	DNA damage response, signal transduction by p53 class mediator	7.424E-10	6	83
	GO:0043516	regulation of DNA damage response, signal transduction by p53 class mediator	2.173E-9	5	44
	GO:0043517	positive regulation of DNA damage response, signal transduction by p53 class mediator	4.584E-9	4	17
	GO:0097190	apoptotic signaling pathway	7.241E-9	10	715
	GO:0071478	cellular response to radiation	8.951E-9	7	225
	GO:0010332	response to gamma radiation	4.104E-8	5	78
	GO:0009314	response to radiation	5.117E-8	9	646
	GO:0045786	negative regulation of cell cycle	8.888E-8	8	483
	GO:1901798	positive regulation of signal transduction by p53 class mediator	9.931E-8	4	35
	GO:0097193	intrinsic apoptotic signalling pathway	2.361E-7	7	363
Cellular components	GO:0061773	eNoSc complex	4.112E-6	2	3
	GO:0140552	TEAD-YAP complex	4.112E-6	2	3
	GO:0033553	rDNA heterochromatin	8.218E-6	2	4
	GO:0000781	chromosome, telomeric region	4.993E-5	4	173
	GO:0005677	chromatin silencing complex	1.237E-4	2	14
	GO:0140513	nuclear protein-containing complex	1.424E-4	8	1386
	GO:0016605	PML body	4.055E-4	3	122
	GO:0098687	chromosomal region	1.439E-3	4	419
	GO:0070310	ATR-ATRIP complex	2.389E-3	1	2
	GO:0032301	MutSalpha complex	2.389E-3	1	2
	GO:0034686	integrin alphav-beta8 complex	2.389E-3	1	2
	GO:0032302	MutSbeta complex	2.389E-3	1	2
	GO:0099126	transforming growth factor beta complex	3.582E-3	1	3
	GO:1902636	kinociliary basal body	3.582E-3	1	3
	GO:0005657	replication fork	3.703E-3	2	76
Molecular pathways	M9703	role of BRCA1, BRCA2 and ATR in Cancer Susceptibility	3.010E-8	4	22
	M39490	DNA IR-damage and cellular response via ATR	1.245E-7	5	81
	M39598	DNA IR-double strand breaks and cellular response via ATM	1.358E-6	4	55
	M648	cell Cycle: G1/S Check Point	1.012E-5	3	28
	137959	BARD1 signalling events	1.128E-5	3	29
	M258	BARD1 signalling events	1.128E-5	3	29
	1270252	molecules associated with elastic fibres	1.384E-5	3	31
	1309108	HDR through Single Strand Annealing (SSA)	2.580E-5	3	38
	1309104	presynaptic phase of homologous DNA pairing and strand exchange	3.016E-5	3	40
	M40049	DNA repair pathways, full network	3.166E-5	4	121
	1309097	sensing of DNA Double Strand Breaks	3.389E-5	2	6
	1270251	elastic fibre formation	3.497E-5	3	42
	1309103	homologous DNA Pairing and Strand Exchange	3.756E-5	3	43
	M39628	integrated cancer pathway	4.310E-5	3	45
	M39518	ATM signalling in development and disease	4.606E-5	3	46

Abbreviations: ATM – ataxia telangiectasia Mutated; ATR – ataxia telangiectasia and Rad3-related; ATRIP – ATR-interacting protein Complex; BARD1 – BRCA1-associated RING domain 1; BRCA1 – breast cancer type 1 susceptibility protein; BRCA2 – breast cancer type 2 susceptibility protein; DNA – deoxyribonucleic acid; HDR – homology directed repair; IR – ionising radiation; MutSalpha – mismatch repair protein MutS alpha; MutSbeta – mismatch repair protein MutS beta; NAD – nicotinamide adenine dinucleotide; PML – promyelocytic leukemia protein; SSA – single-strand annealing; TEAD-YAP – TEA domain transcription factor-Yes-associated protein; eNoSC – embryonic nuclear silencing complex; p53 – tumour protein 53

**Figure 3:**
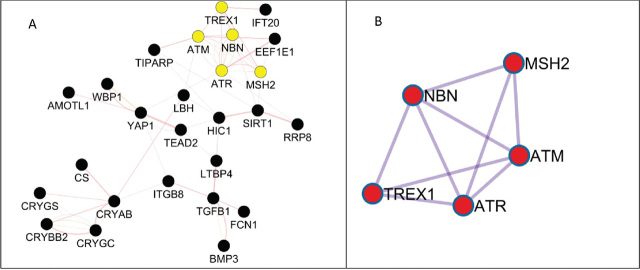
Subnetwork of interconnected genes associated with eye injury caused by ionising radiation (yellow) obtained with MCODE algorithm (*https://apps.cytoscape.org/apps/mcode*): (A) all input genes (n=26); (B) highly interconnected genes

Figure 3A shows all input genes (n=26) forming a subnetwork associated with eye injury caused by ionising radiation, while Figure 3B shows highly interconnected genes within this subnetwork. The most important gene ontologies it identifies are DNA damage checkpoint signalling, DNA integrity checkpoint signalling, and signal transduction in response to DNA damage, which are all part of cellular response to ionising/gamma radiation (Table 4).

**Table 4:** Gene ontology terms linked to the obtained MCODE network [Metascape software (*https://metascape.org*)]

**GO**	**Description**	**Log10(P)**
GO:0010212	response to ionising radiation	−10.5
GO:0071479	cellular response to ionising radiation	−10.4
GO:0071480	cellular response to gamma radiation	−10.4
GO:0000077	DNA damage checkpoint signalling	−12.3
GO:0031570	DNA integrity checkpoint signalling	−12.2
GO:0042770	signal transduction in response to DNA damage	−11.7

Table 5 shows that the identified miRNAs, namely hsa-miR-183 and hsa-miR-589, play a significant role in eye injury caused by ionising radiation, while hsa-miR-892b, hsa-miR-708, hsa-miR-3118, hsa-miR-3166, and hsa-miR-589 play a weaker role.

**Table 5:** miRNAs linked to eye injury caused by ionising radiation (*https://toppgene.cchmc.org*)

**ID**	**Name**	**pValue**	**Input genes**	**Annotated genes**
hsa-miR-892b:mirSVR lowEffct	hsa-miR-892b:mirSVR non-conserved low effect-0.1-0.5	2.811E-7	8	1596
hsa-miR-183:mirSVR highEffct	hsa-miR-183:mirSVR conserved high effect-0.5	1.597E-6	6	853
hsa-miR-708:mirSVR lowEffct	hsa-miR-708:mirSVR non-conserved low effect-0.1-0.5	1.629E-6	7	1376
hsa-miR-3688-3p		2.792E-6	6	940
hsa-miR-3118:mirSVR lowEffct	hsa-miR-3118:mirSVR non-conserved low effect-0.1-0.5	4.640E-6	7	1613
hsa-miR-19a-3p		6.859E-6	6	1100
hsa-miR-19b-3p		7.002E-6	6	1104
hsa-miR-590-5p		1.105E-5	4	317
hsa-miR-3166:mirSVR lowEffct	hsa-miR-3166:mirSVR non-conserved low effect-0.1-0.5	1.146E-5	7	1853
hsa-miR-589:mirSVR highEffct	hsa-miR-589:mirSVR non-conserved high effect-0.5	1.182E-5	7	1862
hsa-miR-548ap-3p		1.281E-5	6	1228
hsa-miR-548t-3p		1.281E-5	6	1228
hsa-miR-548aa		1.281E-5	6	1228
hsa-miR-21-5p		1.455E-5	4	340
hsa-miR-19b:PITA	hsa-miR-19b:PITA TOP	1.615E-5	5	741

Non-conserved miRNAs: miRNAs specific to particular species or closely related groups, contrasting with widely preserved conserved ones. Conserved miRNA: miRNAs highly preserved across diverse species, exhibiting similar sequences and functions, crucial for gene regulation

## DISCUSSION

### Extracted genes

The CTD database listed several diseases/conditions associated with eye injury caused by ionising radiation, including cataract myopathy, alpha-b crystallinopathy, retinal vasculopathy, retinal diseases, dry eye syndromes, and ocular coloboma. These diseases/conditions are linked to six genes, namely *ATM*, *CRYAB*, *SIRT1*, *TGFB1*, *TREX1*, and *YAP1*.

The *ATM* gene is part of the cellular response to DNA damage and plays a critical role in response pathway. ATM protein kinase regulates many signalling pathways by phosphorylating and controlling its substrates, a process whose failure results in genome-wide instability (29).

The *CRYAB* gene encodes a protein called alpha-crystallin B. *CRYAB* binds to crystallins and prevents them from aggregating, while alpha-crystallin B acts as an intracellular chaperone that counteracts oxidative stress-induced damage and apoptosis (30). Crystallins are abundant in the lens and to a smaller degree in other cells, including the retina. They are involved in cytoplasmic organisation and complex molecular mechanisms that regulate cell architecture and function (31). For the lens to be transparent, crystallins must remain densely packed (32), or else mutations increase the risk of developing cataracts (33).

The *SIRT1* gene encodes a protein called Sirtuin 1, which is an epigenetic regulator involved in DNA repair (34) but also in a variety of biological functions such as metabolic regulation, cell maintenance, optimal ageing, and tumorigenesis. It is also active in apoptosis and cell proliferation in reaction to various stressors and metabolic imbalances (35, 36).

The *TGFB1* gene encodes a protein called transforming growth factor beta 1 (TGF-β1), involved in the regulation of cell growth and often associated with anti-proliferative effects (37).

The *TREX1* gene encodes a protein called three prime repair exonuclease 1, whose primary function is to degrade cytoplasmic single-stranded DNA or mispaired 3′ termini of DNA duplexes (38). Mutations in this gene have been associated with the development of age-related cataracts (39).

Finally, the *YAP1* gene encodes a protein called yes-associated protein 1, important for the regulation of cell proliferation (40).

### Molecular functions, biological processes and molecular pathways

As expected, some of the identified molecular functions were related to DNA repair mechanisms (MutLalpha complex binding and mismatch repair complex binding), while others were related to protein binding and enzymatic activities (i.e., protein-containing complex binding). Other functions could be characterised as metabolic processes (citrate synthase activity and citrate (Si)-synthase activity) or post-translational modifications of proteins (protein-propionyllysine depropionylase activity).

On the other hand, the list of biological processes (including those obtained with the MCODE algorithm) was focused on DNA damage and more specifically on the role of the p53 signalling pathway in regulating DNA repair. Ionising radiation can penetrate tissues, disrupt the DNA helix, and cause breaks in one or both strands (41). The resulting DNA damage triggers a cascade of cellular responses, including DNA repair mechanisms and cell cycle checkpoints. If the damage is severe or remains unrepaired, it can lead to genome instability, mutations, cell death, or other adverse outcomes (42). In the context of eye injury caused by ionising radiation, such DNA damage can affect the integrity and function of ocular cells, and if a sufficient number of cells is affected, it can lead to the functional impairment of the lens. As the DNA damage accumulates in the secondary fibre cells for several months after the initial incident, new structures build up that scatter light (43). Wolf et al. (44) reported that 11 Gy of soft x-irradiation, specifically targeting the head region of mice, induced the development of cortical cataracts within the first month, which progressed to an advanced stage 5–11 months after exposure. Although the initial DNA strand breaks were repaired within 30 minutes, DNA damage was persistent over the first 72 h after irradiation, as indicated by the presence of the DNA adduct 8-hydroxyguanosine (8-OHG) and the DNA repair protein X-ray repair cross-complementing protein 1 (XRCC1). This persistence suggests that DNA repair mechanisms may be overwhelmed by radiation-induced DNA lesions and unable to prevent the development of advanced cortical cataracts.

When DNA is damaged, a cascade of signalling events is triggered, which results in the activation of several proteins involved in cell cycle arrest, DNA repair, and apoptosis (45). Lower radiation doses result in lower damage, which allows better repair and reduces the number of cells stuck in the G1/S phase (46). Namely, DNA damage triggers the checkpoint signalling system to prevent the cell from continuing its cycle until the damage has been repaired. Part of this process is the induction of the ATM, ATR, and Chk1/2 proteins, which start cell cycle arrest and DNA repair (47). DNA double strand breaks trigger the ATM/Chk2 pathway, whereas DNA single strand breaks or complex lesions generally start the ATR/Chk1 pathway (47). Markiewicz et al. (48) reported that double strand breaks got repaired more slowly in mouse lens epithelial cells after exposure to 20 than 100 mGy. As a consequence of changes in cell proliferation and density the lens aspect ratio in treated mice changed 10 months after irradiation (48), which suggests impaired DNA repair and checkpoint activation.

Cellular components associated with eye injury (Table 3) consist of regulator complexes such as eNoSC and chromatin silencing. The eNoSC (energy-dependent nucleolar silencing complex), which includes the *SIRT1* gene, silences rDNA and shields mammalian cells from energy-linked apoptosis (49). Others, such as the ATRATRIP complex, are key players in DNA damage response, triggering repair mechanisms upon radiation exposure (47). Additionally, components like PML bodies and the MutS complexes are involved in DNA repair processes crucial for maintaining genomic integrity (50, 51), while growth factor signalling pathways may improve tissue repair mechanisms post-exposure (53).

As for molecular pathways listed in Table 3, some include cancer susceptibility and some DNA damage and cellular response or double strand breaks and cellular response. After exposing retinal photoreceptor cells to ionising radiation doses of 2, 4, 6, 8, 10, and 20 Gy, Yao et al. (52) found increased phosphorylation levels of Chk1 and p53 downstream the ATM pathway (52), which suggests that these signalling events are part of the cellular response to DNA damage and are necessary for the cell to initiate repair mechanisms or apoptosis.

The *BARD1* signalling pathway involves the BARD1 protein in checkpoint activities and DNA damage response and repair (54), while molecules associated with elastic fibres are involved in the formation and maintenance of connective tissue.

Homology-directed repair through single-strand annealing (SSA) and homologous DNA pairing and strand exchange are involved in the mechanisms of DNA repair (55, 56), while the presynaptic stage of homologous DNA pairing and strand exchange pathway is a specific stage in the process of homologous recombination (57).

### miRNA

The list of miRNAs obtained on the basis of genes involved in eye injury caused by radiation could provide valuable insights into the molecular mechanisms underlying this condition. miRNAs are small non-coding RNAs that play important roles in post-transcriptional regulation of gene expression (58) and pathways, and their dysregulation has been implicated in various diseases, including cancer and neurodegenerative disorders (59). Our study has identified several miRNAs with a high (hsa-miR-183 and hsamiR-589) or low effect (hsa-miR-892b, hsa-miR-708, hsa-miR-3118, and hsa-miR-3166) on the target genes. The (miR)183 cluster microRNAs, i.e., miRs-183, -96, and -182, have closely synchronised expression during development and are necessary for sensory organ maturation. They are particularly abundant in retinal photoreceptors and are light-responsive (60).

### Limitations

Our study demonstrates the potential utility of the proposed toxicogenomics data mining in exploring molecular mechanisms of ionising radiation. However, data mining relies on the reliability and completeness of interactions described in online sources such as the CTD database. Furthermore, the obtained data are based on statistical associations between stressor-gene-disease relationships and do not take into account important factors like the dose-response relationship, exposure route, exposure duration, and individual sensitivity.

## CONCLUSION

This study has identified *ATM*, *CRYAB*, *SIRT1*, *TGFB1*, *TREX1*, and *YAP1* as pivotal in radiation-induced eye injury and potential biomarkers for this condition. Their molecular functions encompass DNA repair mechanisms, enzymatic activities, protein binding, metabolic processes, and post-translational modifications of proteins. The pathways involved in DNA damage response and repair include p53 signalling, cell cycle regulation, cancer susceptibility, and developmental pathways. We have also identified several miRNAs, hsa-miR-183 and hsa-miR-589, in particular, which may have an important role in regulating genes involved in DNA repair and ionising radiation-induced eye injury. Our findings contribute to the understanding of the molecular mechanisms underlying the harmful effects of ionising radiation on the eye and provide potential targets of future research.
